# Transcriptional differentiation driving *Cucumis sativus*–*Botrytis cinerea* interactions based on the Skellam model and Bayesian networks

**DOI:** 10.1186/s13568-021-01296-4

**Published:** 2021-10-20

**Authors:** Qi Zhang, Kaihang Li, Yan Yang, Beibei Li, Libo Jiang, Xiaoqing He, Yi Jin, Guozhu Zhao

**Affiliations:** College of Biological Sciences and Technology, Beijing Forestry University, Qinghua East Rd 35, Haidian District, Beijing, 100083 People’s Republic of China

**Keywords:** Transcriptional differentiation, *C. sativus*–*B. cinerea* interaction, Transcriptome, The Skellam model, Bayesian networks

## Abstract

Robust statistical tools such as the Skellam model and Bayesian networks can capture the count properties of transcriptome sequencing data and clusters of genes among treatments, thereby improving our knowledge of gene functions and networks. In this study, we successfully implemented a model to analyze a transcriptome dataset of *Cucumis sativus* and *Botrytis cinerea* before and after their interaction. First, 4200 differentially expressed genes (DEGs) from *C. sativus* were clustered into 17 distinct groups, and 670 DEGs from *B. cinerea* were clustered into 12 groups. Gene ontology (GO) and Kyoto Encyclopedia of Genes and Genomes (KEGG) enrichment analyses were applied on these DEGs to assess the interactions between *C. sativus* and *B. cinerea*. In *C. sativus*, more DEGs were divided into terms in the molecular function and biological process domains than into cellular components, and 277 DEGs were allocated to 19 KEGG pathways. In *B. cinerea*, more DEGs were divided into terms in the biological process and cellular component domains than into molecular functions, and 150 DEGs were allocated to 26 KEGG pathways. In this study, we constructed networks of genes that interact with each other to screen hub genes based on a directed graphical model known as Bayesian networks. Through a detailed GO analysis, we excavated hub genes which were biologically meaningful. These results verify that availability of Skellam model and Bayesian networks in clustering gene expression data and sorting out hub genes. These models are instrumental in increasing our knowledge of gene functions and networks in plant–pathogen interaction.

**Supplementary Information:**

The online version contains supplementary material available at 10.1186/s13568-021-01296-4.

## Introduction

An increasing number of virulent infectious diseases has been witnessed in the past two decades in natural populations and managed landscapes. In recent years, severe economic losses have been caused by an unprecedented number of fungal and fungal-like diseases in both animals and plants (Fisher et al. 2012). Gray mold, caused by *Botrytis cinerea* is a widespread plant fungal pathogen with a necrotrophic nutritional mode and threatens over 230 plants in species worldwide, including economically important crops such as cucumber (Wang et al. 2020). This polyphagous pathogen has been classified as the second most important phyto pathogen and the global impact of *B. cinerea* on plants and plant products is evident due to their broad host ranges (Abbey et al. 2019; AbuQamar et al. 2017; Gao and Zhao 2017). Due to the increasingly severe economic losses caused by *B. cinerea*, an increasing amount of attention has been paid to necrotrophic plant pathogens over the past decade. Cucumber is susceptible to infection by *B. cinerea* (Yuan et al. 2019), which is among the top five important cucumber pathogens (Yu et al. 2019). It is important to understand the molecular mechanisms underlying host–pathogen interactions in devising strategies to control diseases (Vela-Corcía et al. 2019). For this purpose, many *Botrytis* infection mechanisms have been reported in typical plants (El Oirdi et al. 2011; Hou et al. 2019; Hu et al. 2019; Lakkis et al. 2019; Petrasch et al. 2019; Tian et al. 2018; Zhu et al. 2019).

Technological advances facilitate the collection of gene sequencing, gene expression, proteomic, and metabolomic data. The combination of these technologies can yield more information about the mechanisms of plant resistance to pathogens and pathogen–plant infection mechanisms. Transcriptome sequencing is widely implemented to measure the levels of transcripts expressed across various treatments (Kong et al. 2015; Liu et al. 2018; Xiong et al. 2018). Sophisticated statistical modeling offers another way for investigating disease dynamics at multiple biological scales. In addition, it complements and extends the knowledge obtaining from experimental tools (Kirschner and Linderman 2009). Genes divided into the same group may have similar features by cluster analysis, which help us explore the gene functions and networks (Eisen et al. 1998; Ramoni et al. 2002; Sturn et al. 2002) .

However, most model-based cluster analysis approaches have their drawbacks. The Skellam model parameters are estimated by the hierarchical EM algorithm. Skellam modeling is more biologically relevant by comparing with k-means and self-organization mapping (Jiang et al. 2014) reported a Skellam modeling method which grouped genes into different clusters by the patterns of gene expression under different conditions. Therefore, Skellam modeling represents a valuable method to group gene expression data from transcriptome sequencing and enhance our knowledge of gene functions and networks.

The aim of this study was to apply the Skellam framework to explore and cluster co-expression patterns of genes derived from *C. sativus* and *B. cinerea*. We found that the Skellam model was capable of identifying and clustering co-expression models of genes among varied treatments. Moreover, our results will offer insights into the mechanisms of *C. sativus*–*B. cinerea* interactions.

## Materials and methods

### Transcriptome sequencing data

*Botrytis cinerea* is one of the most common crop pathogens. Here, we used *C. sativus *L. (obtained from the Institute of Vegetables and Flowers, Chinese Academy of Agriculture Science) as the host and *B. cinerea* strain B05.10 (provided by China General Microbiological Culture Collection Center) as the pathogen to assess their interaction. Transcriptome sequencing data were obtained from our previous study (Kong et al. 2015). Considering *C. sativus* and *B. cinerea* as an interconnected system, transcriptome sequencing was conducted using infected *C. sativus* leaves, and pure cultured *C. sativus* and pathogen were measured in the same sets. Differential expression between control and treated samples was analyzed by the Bioconductor software package edgeR (McCarthy et al. 2012). A false discovery rate of 0.05 was set as the threshold for significantly different expression.

### Mixture model-based likelihood

The model design followed the method of our previous study (Jiang et al. 2014). Suppose in a transcriptome dataset we measure the organism for reads of n genes with two treatments (1 and 2), and expression reads of gene *i* are described as *X*_*i*_ and *Y*_*i*_, respectively. Briefly, the joint likelihood of the expression data ${z}_{i}=({X}_{i}-{Y}_{i})$ of n genes is written as

1 $$L(\varTheta\vert z)={\prod_{i=1}^n}\left[{{\pi}_1}{f_1}(z_i)+\cdots+{{\pi}_J}{f_J}(z_i)\right],$$

where $\varTheta$ are unknown parameters, ${\pi}_{j}$ is the probability of group $j(j=1,\ldots ,J)$ among the total genes, and ${f}_{j}({z}_{i})$ is the density function of two expression difference values for gene $i$ belonging to group $j$ in the two treatments.

If the two variables are expressed as one dependent random variable, ${\text{z}}_{i}={U}_{1}-{U}_{2}$, the Skellam distribution of ${\text{z}}_{i}$ for gene $i$ is described by a joint probability density function, expressed as

2 $${f_j}\left(Z={z_i}\vert{\varLambda_j}\right)=\text{exp}\left(-({\theta_{j1}}+{\theta_{j2}})\right){\theta_{j1}^{z_i}{\sum_{k=max(0,{-z_i})}^\infty}}\frac{{{({\theta}_{j1}}{\theta_{j2}})}^k}{({z_i}+k)!k!},$$

where $\theta_{j1}$ and $\theta_{j2}$ are the mean expression values of genes which belong to group $j$ in treatments 1 and 2, respectively, with the two parameters arrayed in ${\Lambda_J}=({\theta_{j1}},{\theta_{j2}})$. Here, ${f}_{j}\left({z}_{i}\right)$ in mixture model (1) is specified by ${f_j}(Z={z_i}\vert{\varLambda_j})$.

### Implementation of the EM algorithm

The maximum-likelihood estimates were computed by implementing the EM algorithm. In the E step, the conditional expectation of ${X}_{i}$ was calculate by

$$s_{j\vert i}^{(t)}=E\left(X_{j\vert i}\vert {z_i},{\Lambda_j^{(t-1)}}\right)$$

$$=\sum_{x=0}^\infty\frac{x\times\sum_{j=1}^J{\pi_j^{(t-1)}}{f_j}(X_{j\vert i}=x){f_j}(Y={x-z_i})}{\sum_{j=1}^J{\pi_j^{(t-1)}}{f_j}(Z={z_i})}$$

3 $$=\frac{\sum_{j=1}^J{\theta_{j1}^{(t-1)}\pi}_j^{(t-1)}f_j(z_i-1\vert{\Lambda}_j^{(t-1)})}{\sum_{j=1}^J\pi_j^{(t-1)}f_j\left(z_i\right|{\Lambda}_j^{(t-1)})},$$

where ${f}_{j}^{*}$ is defined in (2). The posterior probability of gene $i$ was calculated which belongs to group $j$,

4 $$\omega_{j\vert i}^{(t)}=\frac{\pi_j^{(t-1)}f_j\left(z_i\right|{\Lambda}_j^{(t-1)})}{\sum_{j=1}^J{\pi_j^{(t-1)}}f_j(z_i|{\Lambda}_j^{(t-1)})},$$

In the M step, the estimates of parameters ${\pi }_{j}$ and ${{\Lambda }}_{j}$ was obtained by

5 $${\pi}_j^{(t)}=\frac{\sum_{i=1}^n\omega_{j\vert i}^{(t)}}n,$$

6 $$\theta_{j1}^{(t)}=\frac{\sum_{i=1}^n\omega_{j\vert i}^{(t)}s_{j\vert i}^{(t)}}{\sum_{i=1}^n\omega_{j\vert i}^{(t)}},$$

7 $$\theta_{j2}^{(t)}=\theta_{j1}^{(t)}-\frac{\sum_{i=1}^n\omega_{j\vert i}^{(t)}z_i}{\sum_{i=1}^n{\omega_{j\vert i}^{(t)}}},$$

The E and M steps are iterated between Eqs. (3–7) until the estimates of the unknown parameters converge to stable values. The estimates obtained this way are the maximum likelihood estimates (MLEs) of the parameters.

### Optimization of the number of groups

For a given number of clusters J, we calculated the likelihood L by (1) and the BIC by − 2 log(L) + J log(n), where n is the number of genes in the model. A low value of BIC corresponds to an optimal number of clusters.

### Hypothesis tests

For a given group $j$, whether its genes are differently expressed between the two treatments can be tested by testing

8 $${H_0}{:}{\theta_{j1}}={\theta_{j2}}\;\text{vs}.\;{H_1}{:}{\theta_{j1}}\neq{\theta_{j2}}\;{\forall_j}=1,\dots, J.$$

If the ${H}_{0}$ is accepted, this means that group of genes expressed between two treatments is stable. Otherwise, they show different amounts of expression before and after interaction, in which case they can be used as a predictor of interaction-induced changes. For a pair of groups, we further tested whether they interacted with each other to determine interaction-induced changes.

### Enrichment analysis

The GO enrichment analyses of genes were tested using the hypergeometric distribution and the definition of the hypergeometric distribution is as follows,

9 $$P(z/T,\;S,\;n)=\frac{\left({\frac Sz}\right)\left({\frac{T-S}{n-z}}\right)}{\left({\frac Tn}\right)},$$

where *T* and *n* are the total numbers of genes and DEGs, respectively, and *S* and *z* are the numbers of genes and DEGs that belong to a certain functional category, respectively. The significant GO categories were selected with false discovery rate less than 0.05. Hypergeometric distribution method was also used to examine the statistical enrichment of DEGs in the KEGG pathways (Abbey et al. 2019; Young et al. 2010).

### Gene regulatory network reconstruction

Gene regulatory network are visual representations of mechanisms that make up the functioning of an organism under given conditions. The methods that were proposed and developed include analyses based on correlations, ordinary or partial differential equations, and Bayesian networks. Bayesian networks are a promising tool for inferencing gene regulatory network (Vignes et al. 2011). In this study, we considered that this approach was suitable for the experimental design and data property. The structure and parameters of the underlying graph were estimated by a score-based structure learning algorithm similarly to what was done in previous reports (Scutari and Denis 2014; Vignes et al. 2011).

## Results

### Differential expression analysis

A false discovery rate of 0.05 was set as the threshold for significantly different expression. In order to understand the response of *C. sativus* to *B. cinerea* infection, GO analysis was implemented to the above DEGs, and enrichment analysis was applied based on the hypergeometric distribution, using a false discovery rate (FDR) of < 0.05 as the cutoff.

In *C. sativus*, more DEGs were divided into terms in the molecular function and biological process domains than to cellular component terms. The dominant terms in each domain were “phosphotransferase activity”, “oxidation–reduction process”, and “integral to membrane”, respectively (Additional file 7: Figure S1A). The most significantly enriched GO terms in the molecular function domain included “phosphotransferase activity—alcohol group as acceptor” (GO:0016021), “GTP binding” (GO:0015979), “ATP binding” (GO:0005576), “protein tyrosine/serine/threonine phosphatase activity” (GO:0005506), and “heme binding” (GO:0009765). The most significantly enriched GO terms in the biological process domain included “oxidation–reduction process” (GO:0055114), “negative regulation of transcription”, “DNA-dependent” (GO:0009734), “protein phosphorylation” (GO:0004601), “oxidation–reduction process” (GO:0009522), and “carbohydrate transport” (GO:0004497) (Additional file 1: Table S1).

In *B. cinerea*, more DEGs were divided into terms in the biological process and cellular component domains than to molecular function terms. The dominant terms in each domain were “transport”, “cytosol”, and “hydrolase activity”, respectively (Additional file 7: Figure S1B). The most significantly enriched GO terms in the molecular function domain included “hydrolase activity” (GO:0005975), “oxidoreductase activity” (GO:0004553), “TBP-class protein binding” (GO:0003868), “purine nucleobase transmembrane transporter activity” (GO:0070884; GO:0046355), and “RNA polymerase I activity” (GO:0045461). The most significantly enriched GO terms in the biological process domain included “transport” (GO:0030248), “oxidation–reduction process” (GO:0055114; GO:0016812), “metabolic process” (GO:0016491; GO:0007346), “mitochondrial transport” (GO:0008864), “vesicle-mediated transport” (GO:0030245), and “methylation” (GO:0004076) (Additional file 2: Table S2).

To further clarify the functions of DEGs, they were mapped to KEGG terms to identify genes involved in significantly enriched biosynthetic or signal transduction pathways in *C. sativus* and *B. cinerea*. 277 DEGs were assigned to 19 KEGG pathways in *C. sativus* (Additional file 3: Table S3). The top five significantly enriched biosynthetic pathways included “phenylpropanoid biosynthesis”, “photosynthesis”, “biosynthesis of antibiotics”, “fatty acid elongation”, and “valine, leucine, and isoleucine degradation” (Additional file 8: Figure S2A). The pathway involving the highest number of DEGs was “biosynthesis of antibiotics” (86; 31.05%), followed by “phenylpropanoid biosynthesis” (53; 19.13%), “starch and sucrose metabolism” (35; 12.64%), “pentose phosphate pathway” (22; 7.94%), and “glycine, serine, and threonine metabolism” (14; 5.05%). Therefore, we considered the DEGs involved in these pathways as candidates associated with *C. sativus* susceptibility to *B. cinerea*.

In *B. cinerea*, 150 DEGs were assigned to 26 KEGG pathways (Additional file 4: Table S4). Among these, “starch and sucrose metabolism”, “pentose and glucuronate interconversions”, “cyanoamino acid metabolism”, “biosynthesis of antibiotics”, and “phenylpropanoid biosynthesis” were the top five most significantly enriched pathways (Additional file 8: Figure S2B). The pathway involving the highest number of DEGs was “biosynthesis of antibiotics” (27; 18.00%), followed by “starch and sucrose metabolism” (16; 10.67%), “pentose phosphate pathway” (11; 7.33%), “phenylpropanoid biosynthesis” (9; 6.00%), “cyanoamino acid metabolism” (7; 4.67%), and “glyoxylate and dicarboxylate metabolism” (7; 4.67%).

### Clustering using the Skellam model

**Fig. 1:**
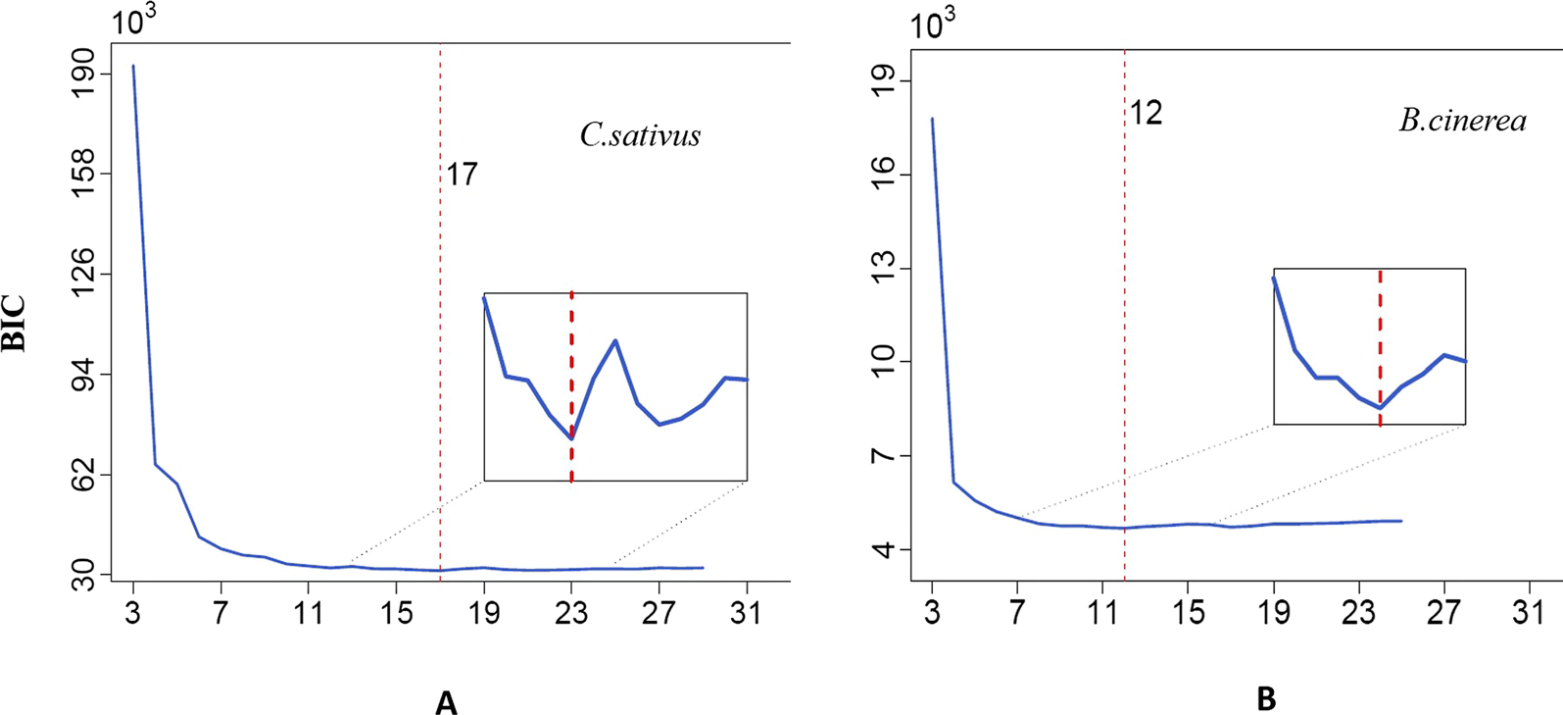
Plot of BIC values over the number of groups calculated from the transcriptomic data. **A** *C. sativus*; **B** *B. cinerea*

**Fig. 2:**
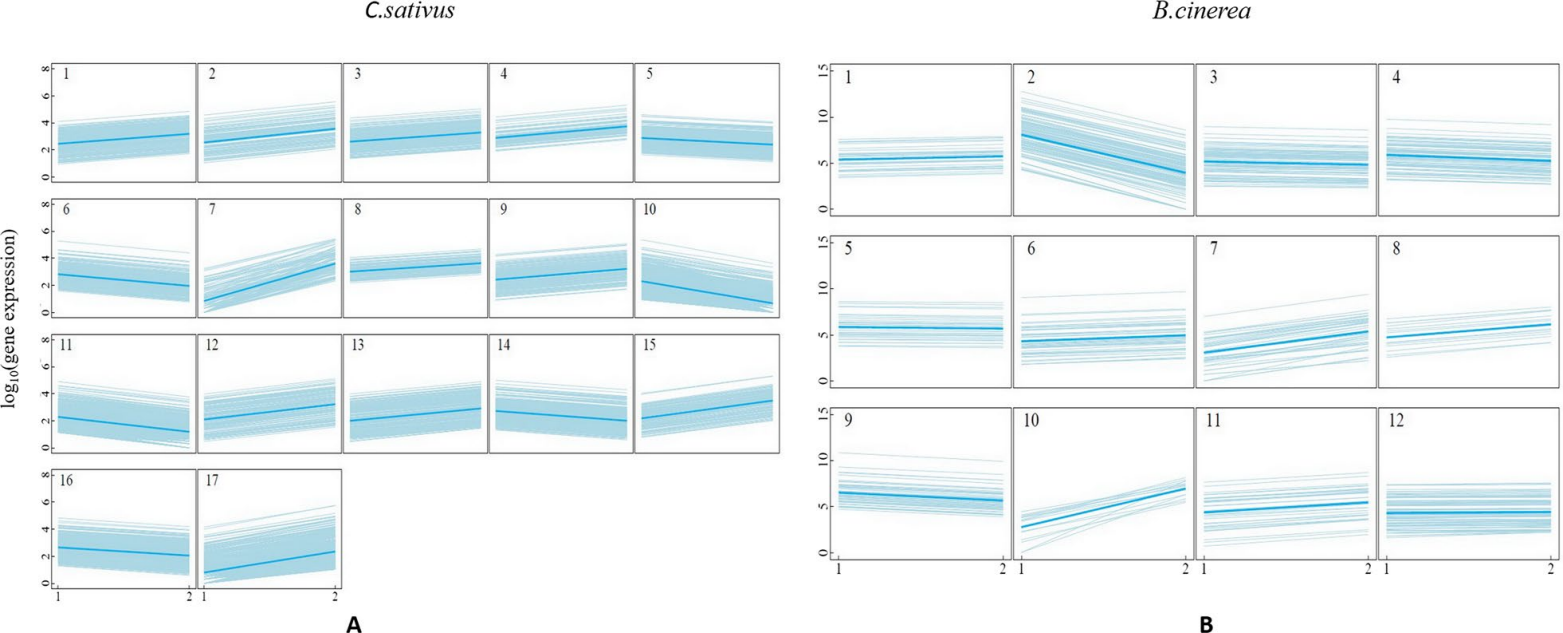
Differentiation patterns of genes from distinct groups. **A** There are 17 groups expressed in *c. sativus*; **B** there are 12 groups expressed in *B. cinerea.* In each group, the mean expression curve is indicated by a thick line over expressions curves of individual genes (thin lines)

**Fig. 3:**
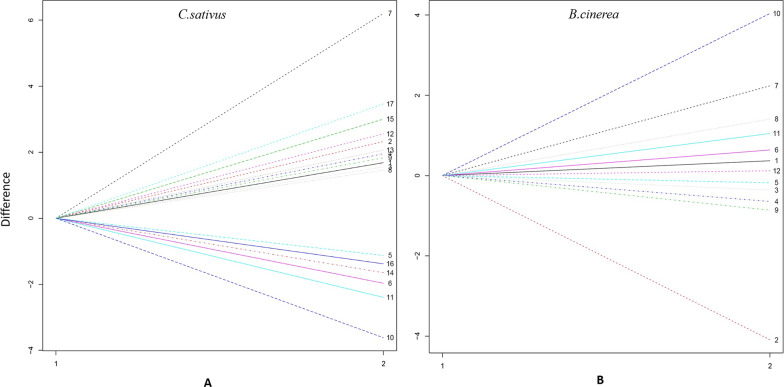
Relative differences among gene expression curves of different groups expressed in **A** *C. sativus* and **B** *B. cinerea*

The Skellam model was used to cluster RNA genes into distinct groups. Because it incorporates sample size information, we used the Bayesian information criterion (BIC) as the model-selection criterion. First, we clustered 4200 differentially expressed genes (DEGs) in *C. sativus* into distinct groups. From the plot of the BIC against the group numbers, all the DEGs are categorized into 17 distinct groups (Fig. 1A). We had illustrated the mean expression in each group of *C. sativus* and these 17 groups displayed differential levels in expression (Fig. 2A and Additional file 5: Table S5). Figure 3A plotted the pattern of the *C. sativus* gene expression differences before and after fungal infection, which showed that DEGs in 11 groups were up-regulated, whereas those in 6 groups were down-regulated. The gene groups were not parallel and different patterns of gene expression plasticity was exhibited in response to environmental changes from an uninfected state to an infected one. Subsequently, based on the BIC values under different numbers of clusters, 670 DEGs in *B. cinerea* were clustered into 12 groups (Fig. 1B). The mean expression values in each group of *B. cinerea* were showed in Fig. 2B and Additional file 6: Table S6. The pattern of pathogen gene expression differences before and after host infection, in which DEGs in 7 groups were up-regulated, whereas those in 5 groups were down-regulated (Fig. 3B).

### Plasticity expression pattern

Of these 17 groups in *C. sativus*, gene expression levels from groups 1, 2, 3, 4, 7, 8, 9, 12, 13, 15, 17 (accounting for nearly 42.5% of genes) were clearly up-regulated after *B. cinerea* infection. Nearly 50% of genes (groups 6, 10, 11, 14, 16) were clearly down-regulated and gene expression levels from group 5 (about 9.4%) tended to be slightly down-regulated. In group 7, the most significantly enriched GO term responded to “oxidative stress” (GO:0006979), indicating that the plant reacted to pathogen infection. GO term “cell wall” (GO:0005618) was significantly enriched in group 11 and term “photosystem” was significantly enriched in group 14. In *B. cinerea*, of these 12 groups, only the mean expression values of group 2 (about 18.4%) were clearly down-regulated after infecting *C. sativus*. Approximately 40.15% of genes from groups 3, 4, 5, 9 were slightly down-regulated. Genes in other groups are up-regulated after infecting *C. sativus*. Hypothesis tests were performed to examine whether each cluster of genes expressed significantly differently between the two treatments and determined whether a particular pair of gene groups interacted with the environment. Plasticity gene expression was statistically significant (P < 0.05). This indicated that DEGs tended to obvious changes in response to *B. cinerea* infection. All pairs of gene clusters displayed significant gene-environment interactions (P < 0.05).

### Gene regulatory network

The core-periphery structure is a vital feature of many biological networks, including protein-protein interaction networks as well as gene regulatory and metabolic networks (Csermely et al. 2013). In this study, we constructed networks of genes that interacted with each other to screen hub genes based on a directed graphical model known as Bayesian networks. Through a detailed GO analysis, we detected hub genes which were biologically meaningful.

**Fig. 4:**
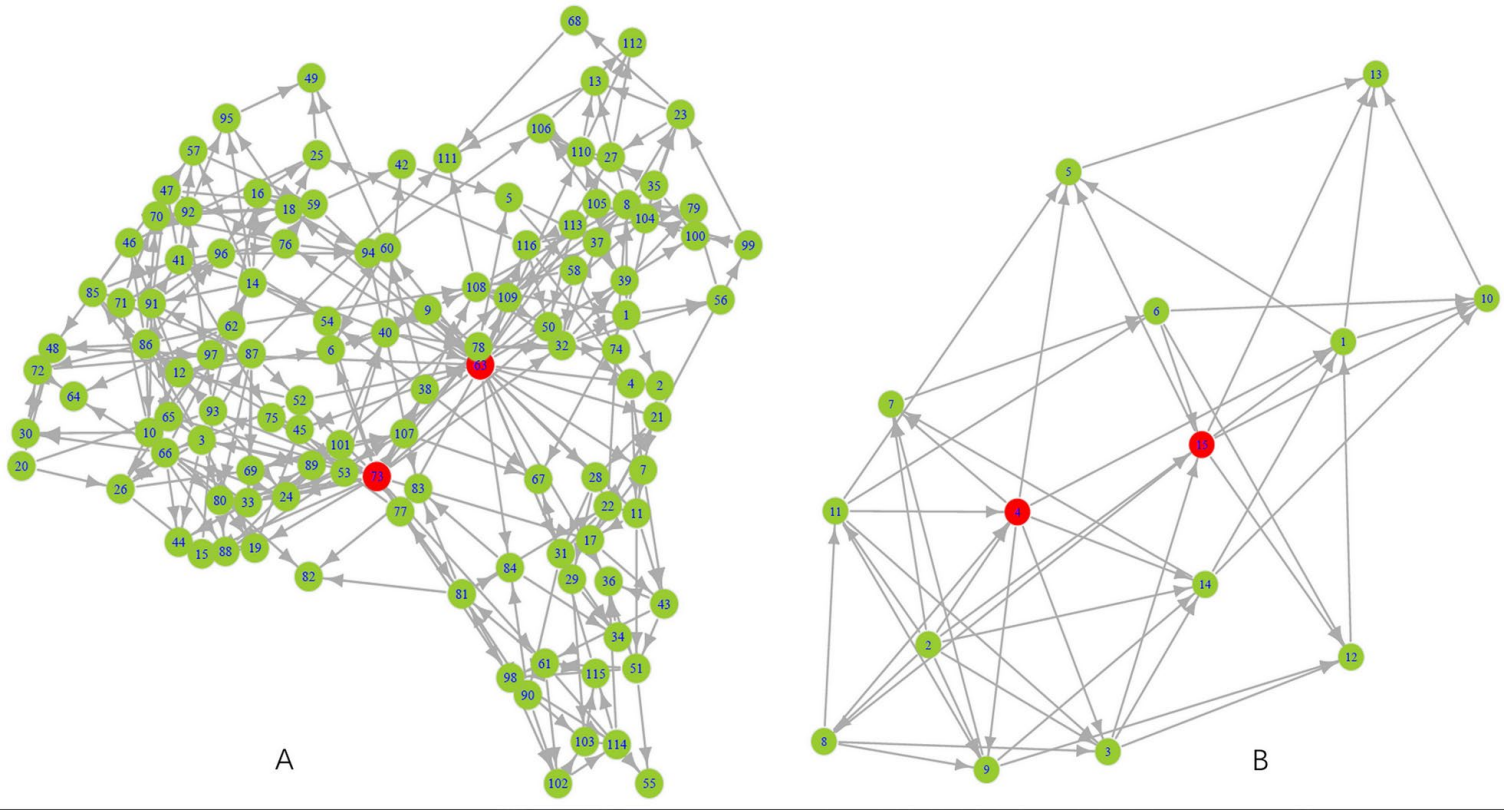
The gene regulatory network by Bayesian networks in **A** group 7 of *C. sativus* and **B** group 10 of *B. cinerea*

For example,the gene regulatory network of group 7 in *C. sativus* was shown in Fig. 4A. All the 116 genes were displayed in green (Additional file 5: Table S5), in which two in red were No. 63 (Csa5G285030, Proteinase inhibitor) and 73 (Csa1G265640, Uncharacterized protein). They were two hub genes detected by the Bayesian networks. Csa5G285030 was enriched in “response to wounding” (GO:0009611), which might be involved in response to stress such as wounding and pathogens. Group 12 contained 149 genes in the network (Additional file 5: Table S5), in which No. 29 (Csa2G075440) was screened as a hub gene. Csa2G075440 was annotated as “disease resistance protein RPS2” in KEGG orthology and enriched in the pathway of “plant–pathogen interaction” (Bent et al. 1994). In *B. cinerea*, the mean expression values of group 10 were clearly up-regulated after infecting *C. sativus*. There were 15 genes in this group, in which No. 4 (B0510_3699) was identified as one of the hub genes (Fig. 4B). The gene probably encodes 1,4-beta-d-glucan cellobiohydrolase which participates in regulating the hydrolase activity or hydrolyzing *O*-glycosyl compounds (GO:0004553) for pathogens to invade plant cells or exploit the polysaccharides of plant cell walls (Additional file 6: Table S6) (Kong et al. 2015).

## Discussion

Plant–pathogen interactions are a topic of scientific interest. With the advent of deep-sequencing-based transcriptome sequencing, the expression levels of transcripts can be precisely measured in any tissue (Wang et al. 2009). Pathogen gene expression programs in answer to the host environment and host gene expression in response to pathogens can be monitored more easily by this method. It is crucial to measure the dynamic behavior of gene expression for interpreting the genetic mechanisms of host–pathogen interactions.

By transcriptome sequencing, we previously had investigated whole-transcriptome profile changes in *C. sativus* and *B. cinerea* before and after infection. However, in order to analyze the transcriptome sequencing results better, a powerful statistical method is needed. Here, we present a computational model combined with transcriptome sequencing data to investigate *C. sativus*–*B. cinerea* interactions.

As a useful tool, cluster analysis can help us analyze gene expression derived from different gene expression patterns. Using traditional methods, gene clustering is only performed by their expression at single points or their joint expression at multiple points and doesn’t consider how different conditions affect the expression of genes. The Skellam model treats the co-expression of genes under different conditions as a system and integrates the capacity of a cross-treatment genes to co-respond to environmental changes into clustering procedures, for better understanding the gene responses to certain external conditions (Jiang et al. 2014). Nevertheless, most existing model-based cluster analysis approaches have not adapted to the particular properties of transcriptome sequencing data or do not consider extraordinary experimental conditions. However, the current Skellam model allows for the classification of two reaction norms in response to an environmental signal. To model successive changes in gene expression in response to environmental stimuli, the extended statistical sekllam model such as the bivariate Skellam, multivariate Skellam and Poisson–Skellam probability distribution are required (Akpoue and Angers 2016; Bulla et al. 2015; Gan et al. 2015; Lu et al. 2015; Wang et al. 2014).

Skellam modeling has been used successfully to cluster genes from early *Arabidopsis thaliana* embryos into groups (Jiang et al. 2014). For example, group 9 was related to proteins like ATP-involved ATP synthase 9. Group 8 was associated with proteins such as pathogenesis-related thaumatin-like protein. Moreover, during the initial stages, both the maternal and paternal genomes were active with essentially equivalent contributions to the embryonic transcriptome; however, the activated gene sets differed. Meanwhile, as mentioned above, Jiang et al. (2014) clustered the transcriptome sequencing dataset of early *A. thaliana* embryos by the level of maternal and paternal genome contributions. The validation of this model has also been performed by simulation studies.

Plant–pathogen interactions are complicated processes which cause a series of molecular responses at various expression levels. Compared with our previous study, the GO enrichment analysis in the present study showed that several of the same GO terms were among the top 10 significantly enriched terms involved in *B. cinerea* infection, including hydrolase activity, metabolic process, oxidation–reduction process, and oxidoreductase activity (Additional file 2: Table S2). In *C. sativus* resistance, only one of the same GO terms, oxidation–reduction process, was significantly enriched (Additional file 1: Table S1). Meanwhile, the KEGG enrichment analysis of *C. sativus* showed that only three of the most enriched pathways (“photosynthesis”, “valine, leucine, and isoleucine degradation” and “pentose phosphate pathway”) were the same as those identified in our previous research (Additional file 3: Table S3). In *B. cinerea*, several KEGG pathways, such as “starch and sucrose metabolism”, were the same as those identified in our previous study (Additional file 4: Table S4). Some significantly enriched biosynthetic pathways, including “phenylpropanoid biosynthesis”, “photosynthesis”, “valine, leucine and isoleucine degradation”, “starch and sucrose metabolism” and “zeatin biosynthesis”, were in agreement with the major pathways involved in plant–pathogen interactions identified in a similar study (Liu et al. 2016). A previous report found that some genes involved in the phenylpropanoid pathway were induced during the compatible interaction between *Lactuca sativa* and *B. cinerea* (De Cremer et al. 2013). The response of susceptible plants was slower and milder than that of resistant plants, although this metabolic pathway was activated in both susceptible and resistant plants (Tan et al. 2015). Meanwhile, “photosynthesis” was the second-most significantly enriched pathway, consistent with our previous study, which confirms that photosynthesis plays an important role in pathogen resistance (Kong et al. 2015).

Through a detailed network analysis, we can better chart a picture of the mechanistic regulation of genes for pathogens infection and stress tolerance in plants. Several hub genes have been detected by Bayesian networks such as genes encoded “disease resistance protein RPS2” in the plant. RPS2 confers resistance to strains of the bacterial phytopathogen *Pseudomonas syringae* carrying the avirulence genes avrRpt2 (Bent et al. 1994; Leister et al. 1996). 1,4-Beta-d-glucan cellobiohydrolase was identified as a hub gene in the pathogen, which meant for pathogens to invade the polysaccharides of plant cell walls they must secrete enzymes to disassemble cell wall polysaccharides (Kong et al. 2015; Zhu et al. 2017).

It is crucial to measure the dynamic behavior of gene expression for explaining the genetic mechanisms of host–pathogen interactions; however, most studies of gene expression based on transcriptome sequencing have been performed in a static state. The Skellam distribution and Bayesian networks facilitate us to elucidate a more precise characterization of host–pathogen interactions and co-evolution. Finally, it is necessary to integrate the multivariate Skellam distribution and Bayesian networks to support further investigations using more sophisticated statistical models.

## Supplementary Information

**Additional file 1: Table S1.** GO enrichment analysis of all differential expression gene in *C. sativus*.

**Additional file 2: Table S2.** GO enrichment analysis of all differential expression gene in *B. cinerea*.

**Additional file 3: Table S3.** Significantly enriched KEGG pathways of DEGs in *C. sativus*.

**Additional file 4: Table S4.** Significantly enriched KEGG pathways of DEGs in *B. cinerea*.

**Additional file 5: Table S5.** Clustering of all differential expression gene in *C. sativus.*

**Additional file 6: Table S6.** Clustering of all differential expression gene in *B. cinerea.*

**Additional file 7: Figure S1.** GO enrichment analysis of differential expression genes in (A) *C. sativus* and (B) *B. cinerea*. The colors reflect different domains and circle areas reflect the number of genes associated to a given GO term.

**Additional file 8: Figure S2.** KEGG enrichment analysis of differential expression genes in (A) *C. sativus* and (B) *B. cinerea.* The circle areas reflect the number of genes associated to a given KEGG term.

## Data Availability

Data sequences: the raw sequence data generated in this study were deposited in the NCBI Gene Expression Omnibus under Accession No. GSE72191. The computer codes are available online at https://github.com/lenahe2006.

## Abbreviations

EM: Expectation-maximization
DEGs: Differentially expressed genes
GO: Gene ontology
KEGG: Kyoto Encyclopedia of Genes and Genomes
ATP: Adenosine triphophate
