# Supplementary material for: Transcriptional differentiation driving Cucumis sativus–Botrytis cinerea interactions based on the Skellam model and Bayesian networks
Source: AMB Express. 2021 Oct 20;11:138. doi: 10.1186/s13568-021-01296-4 (PMC8528924; doi:10.1186/s13568-021-01296-4)
Supplement: Supplementary file 3 — Additional file 3: Table S3. Significantly enriched KEGG pathways of DEGs in C. sativus. [file 13568_2021_1296_MOESM3_ESM.docx]

| **Pathway** | **No. of DEGs** | **-log10(FDR)** | **Pathway ID** |
| --- | --- | --- | --- |
| Phenylpropanoid biosynthesis | 53 | 10.40077208 | map00350 |
| Photosynthesis | 3 | 3.142768116 | map00195 |
| Biosynthesis of antibiotics | 86 | 3.142768116 | map01130 |
| Fatty acid elongation | 5 | 3.142768116 | map00062 |
| Stilbenoid, diarylheptanoid and gingerol biosynthesis | 1 | 3.142768116 | map00945 |
| Ethylbenzene degradation | 1 | 3.142768116 | map00642 |
| Valine, leucine and isoleucine degradation | 9 | 2.840638677 | map00280 |
| Pentose phosphate pathway | 22 | 2.746468356 | map00030 |
| Geraniol degradation | 5 | 2.649726953 | map00281 |
| Caprolactam degradation | 4 | 2.649726953 | map00930 |
| Benzoate degradation | 5 | 2.159516551 | map00362 |
| Toluene degradation | 3 | 2.043428438 | map00623 |
| Primary bile acid biosynthesis | 4 | 2.043428438 | map00120 |
| Fatty acid degradation | 7 | 2.043428438 | map00071 |
| Glycerolipid metabolism | 11 | 1.867640144 | map00561 |
| Starch and sucrose metabolism | 35 | 1.851932044 | map00500 |
| Zeatin biosynthesis | 4 | 1.37561246 | map00908 |
| Cyanoamino acid metabolism | 5 | 1.37561246 | map00460 |
| Glycine, serine and threonine metabolism | 14 | 1.37561246 | map00260 |

Table S3 Significantly enriched KEGG pathways of DEGs in *C.sativus*
