# Supplementary material for: Transcriptional differentiation driving Cucumis sativus–Botrytis cinerea interactions based on the Skellam model and Bayesian networks
Source: AMB Express. 2021 Oct 20;11:138. doi: 10.1186/s13568-021-01296-4 (PMC8528924; doi:10.1186/s13568-021-01296-4)
Supplement: Supplementary file 4 — Additional file 4: Table S4. Significantly enriched KEGG pathways of DEGs in B. cinerea. [file 13568_2021_1296_MOESM4_ESM.docx]

Table S4 Significantly enriched KEGG pathways of DEGs in *B.cinerea*

| **Pathway** | **No. of DEGs** | **-log10(FDR)** | **Pathway ID** |
| --- | --- | --- | --- |
| Starch and sucrose metabolism | 16 | 6.703397754 | map00500 |
| Pentose and glucuronate interconversions | 11 | 5.240371202 | map00040 |
| Cyanoamino acid metabolism | 7 | 3.907658744 | map00460 |
| Biosynthesis of antibiotics | 27 | 3.595693024 | map01130 |
| Phenylpropanoid biosynthesis | 9 | 3.595693024 | map00940 |
| Glycosphingolipid biosynthesis - ganglio series | 3 | 2.584899387 | map00604 |
| Glycosaminoglycan degradation | 3 | 2.584899387 | map00531 |
| Fructose and mannose metabolism | 6 | 2.470061939 | map00051 |
| Glyoxylate and dicarboxylate metabolism | 7 | 2.419128934 | map00630 |
| Propanoate metabolism | 4 | 2.366246658 | map00640 |
|  |  |  |  |
| Valine, leucine and isoleucine degradation | 5 | 2.286982231 | map00280 |
| Butanoate metabolism | 5 | 2.286982231 | map00650 |
| Aminobenzoate degradation | 5 | 2.227010142 | map00627 |
| Glucosinolate biosynthesis | 2 | 2.227010142 | map00966 |
| Synthesis and degradation of ketone bodies | 2 | 2.227010142 | map00072 |
| Other glycan degradation | 3 | 2.080401063 | map00511 |
| Ubiquinone and other terpenoid-quinone biosynthesis | 1 | 1.664646544 | map00130 |
| Terpenoid backbone biosynthesis | 3 | 1.664646544 | map00900 |
| Sphingolipid metabolism | 4 | 1.664646544 | map00600 |
| Phenylalanine metabolism | 3 | 1.664646544 | map00360 |
| Galactose metabolism | 5 | 1.664646544 | map00052 |
| Glycine, serine and threonine metabolism | 5 | 1.445084075 | map00260 |
| Pantothenate and CoA biosynthesis | 4 | 1.414724099 | map00770 |
| Valine, leucine and isoleucine biosynthesis | 3 | 1.347999867 | map00290 |
| Biotin metabolism | 1 | 1.347999867 | map00780 |
| Cysteine and methionine metabolism | 6 | 1.347999867 | map00270 |
