# Supplementary figures and images for: Transcriptional differentiation driving Cucumis sativus–Botrytis cinerea interactions based on the Skellam model and Bayesian networks
Source: AMB Express. 2021 Oct 20;11:138. doi: 10.1186/s13568-021-01296-4 (PMC8528924; doi:10.1186/s13568-021-01296-4)

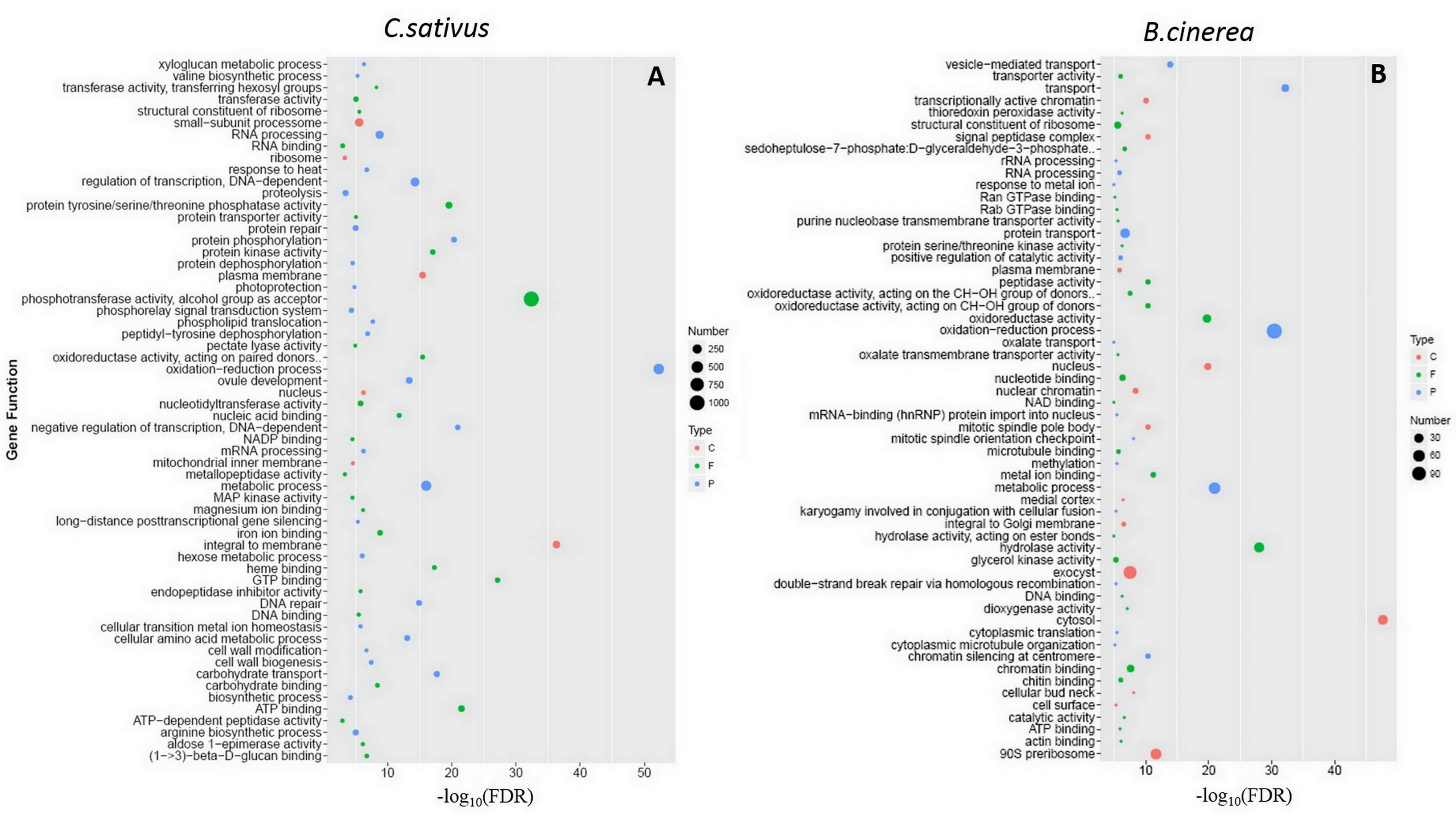

Supplement: Supplementary file 7 — Additional file 7: Figure S1. GO enrichment analysis of differential expression genes in (A) C. sativus and (B) B. cinerea. The colors reflect different domains and circle areas reflect the number of genes associated to a given GO term. [file 13568_2021_1296_MOESM7_ESM.jpg]

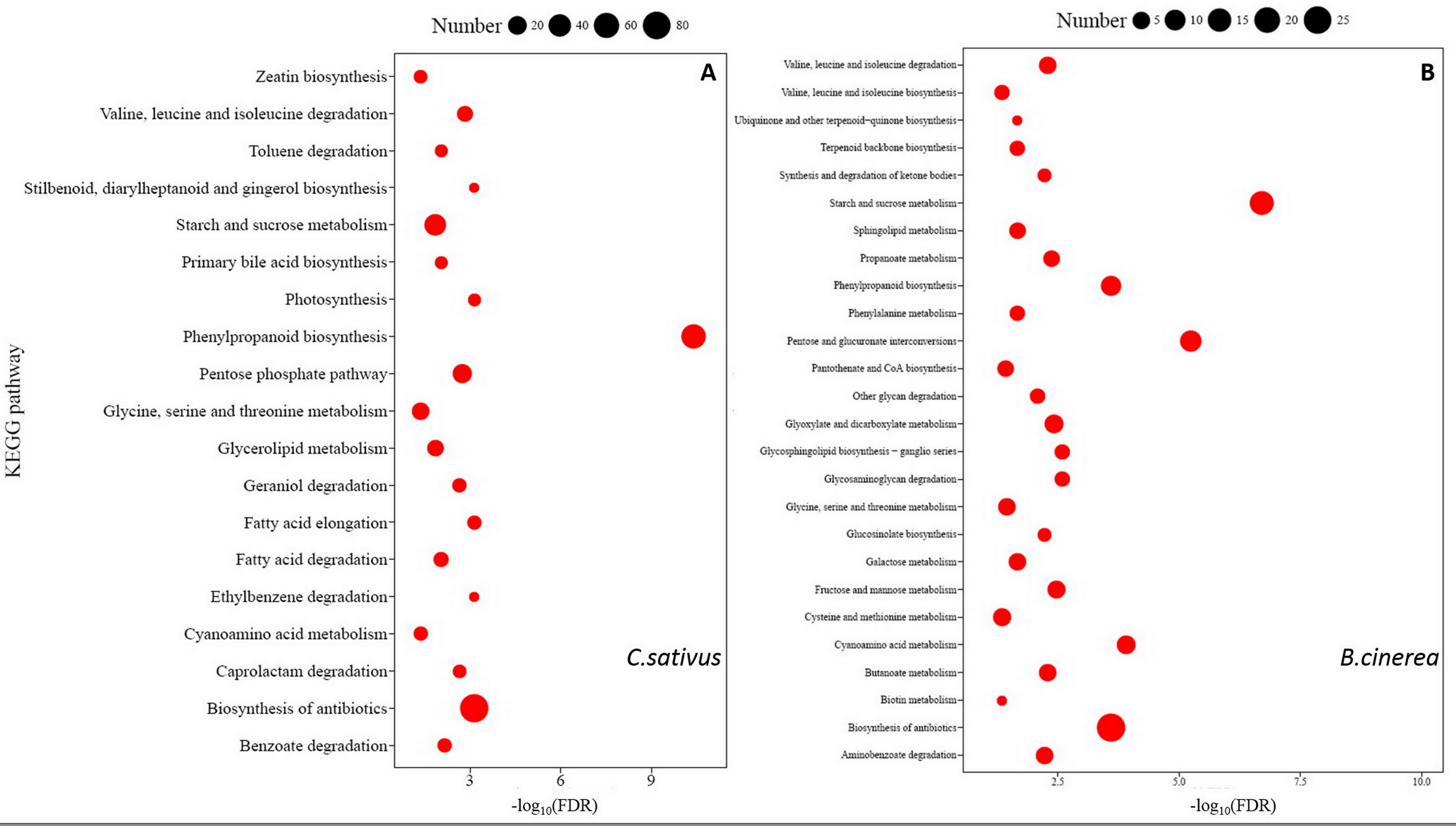

Supplement: Supplementary file 8 — Additional file 8: Figure S2. KEGG enrichment analysis of differential expression genes in (A) C. sativus and (B) B. cinerea. The circle areas reflect the number of genes associated to a given KEGG term. [file 13568_2021_1296_MOESM8_ESM.jpg]
